# Interaction of gallium, indium and vanadyl diacetylcurcumin complexes with lysozyme: mechanistic aspects and evaluation of antiamyloidogenic activity

**DOI:** 10.1098/rsos.230443

**Published:** 2023-11-08

**Authors:** Amin Sahraei, Ahmad Ehsanfar, Fakhrossadat Mohammadi

**Affiliations:** Department of Chemistry, Institute for Advanced Studies in Basic Sciences (IASBS), 444 Prof. Sobouti Boulevard, Gava Zang, Zanjan 45137-66731, Iran

**Keywords:** gallium diacetylcurcumin, indium diacetylcurcumin, vanadyl diacetylcurcumin, amyloid fibrils, lysozyme

## Abstract

Diacetylcurcumin as a derivative of curcumin is a strong nitric oxide (NO) and O_2_^−.^anion scavenger. One strategy to improve stability of curcumin and its derivatives is complexation with metal. In this study, the binding interactions of gallium diacetylcurcumin (Ga(DAC)_3_), indium diacetylcurcumin (In(DAC)_3_), and vanadyl diacetylcurcumin (VO(DAC)_2_) with hen egg white lysozyme (HEWL) have been investigated. The results of fluorescence quenching analyses revealed that In(DAC)_3_ and VO(DAC)_2_ have higher binding affinities than Ga(DAC)_3_ towards HEWL. The interactions of these metal complexes were not accompanied by considerable conformational changes in the tertiary structure of HEWL. Furthermore, the inhibitory effects of these complexes on the amyloid fibrillation of HEWL were confirmed by the thioflavin T fluorescence assays. The kinetic curves of the fibrillation process illustrated that VO(DAC)_2_ has the highest inhibitory activity and In(DAC)_3_ has a significant delaying effect on the formation of amyloid fibrils of HEWL.

## 1. Introduction

Amyloid fibrillation, a common pathological hallmark of several devastating neurodegenerative and metabolic disorders, including Alzheimer's, Parkinson's and Huntington's diseases and type II diabetes, is a biological process in which normally soluble proteins and peptides are converted into long, insoluble and highly ordered aggregates with a high β-sheet content termed as amyloid fibrils [1,2]. According to recently published reports, it can be stated that the consequences of such diseases on human health and welfare would be catastrophic in the near future if novel treatment strategies with high efficacy are not developed. For example, Alzheimer's disease (AD), as a twenty-first century plaque, will affect more than 80 million new cases over a 40-year period from 2010 onwards, and the financial burden was $236 billion in 2016 alone [1,3]. Parkinson's disease affects approximately 2% of individuals over the age of 60, equivalent to about one million people in the United States in 2011, and 50 000 new cases are diagnosed every year [4,5]. Thus, significant efforts are being made worldwide to detect amyloid aggregates, understand the relatively obscure mechanisms of amyloid formation, and find more effective therapeutic agents for amyloid-related disorders using curcumin [6] and its derivatives [7,8], flavonoids like morin and myricetin [9,10], and different kinds of nanomaterials [11–13].

**Figure 1.**
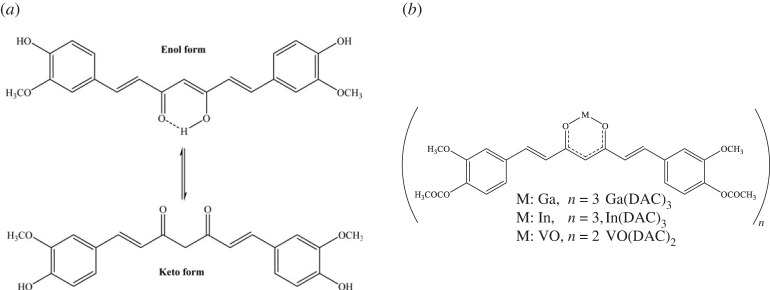
Chemical structures of curcumin (*a*) and its metal-complexed derivatives used in this work.

Curcumin (figure 1), 1,7-bis(4-hydroxy-3-methoxyphenyl)-1,6-heptadiene-3,5-dione (diferuloylmethane), is one of the most well-known natural polyphenolic compounds obtained from the root of the Indian plant (*Curcuma longa*) whose promising biological and medical properties, including antioxidant, anti-inflammatory, antimicrobial, antitumour, antidiabetic and neuroprotective activities has been well established [14–16]. Clinical studies in humans have shown that curcumin is quite safe and non-toxic up to doses of 12 g per day [14,16]. Although instability and poor solubility in aqueous media and rapid metabolism in the gastrointestinal tract have limited the usage of curcumin in clinical diagnosis and treatment, it has been shown that the complexation of curcumin with metals, in addition to tackling the solubility and bioavailability problems, enhances its pharmacological effects [16,17]. Lange *et al*. [18] have recently synthesized a lipophilic Ga^+3^ Schiff base-curcumin complex that binds to amyloid-β (A*β*) plaques in human brain tissue. In an elegant work, Zhang *et al*. [8] have demonstrated that CRANAD-17, a synthetic imidazole-containing curcumin scaffold, considerably reduces A*β* amyloid formation in AD by inhibiting A*β*42 cross-linking induced by copper. Mohammadi *et al*. synthesized and characterized gallium diacetylcurcumin (Ga(DAC)_3_), indium diacetylcurcumin (In(DAC)_3_), and vanadyl diacetylcurcumin (VO(DAC)_2_) (figure 1) and investigated their anti-oxidant capacities and cytotoxic activities [19]. Their results showed that the cytotoxicity effects of Ga(DAC)_3_ and In(DAC)_3_ on mouse lymphoma cells (IC_50_∼25–30 µM, and 20–25 µM, respectively) were similar to that of curcumin (IC_50_∼25–30 µM), whereas, the cytotoxic effect of VO(DAC)_2_ (IC_50_∼7–10 µM) was similar to that of diacetylcurcumin (IC_50_∼10–15 µM). The Trolox equivalent antioxidant assay showed that these complexes have almost the same antioxidant capacity as curcumin.

Hen egg-white lysozyme (HEWL) is a small basic enzyme with a low molecular weight of 14.3 kDa and a high isoelectric point (pI) of 10.7, whose 129 amino acid residues are linked together by four disulfide bridges to form a single polypeptide chain [20,21]. On one hand, the ease of HEWL fibril formation under various experimental conditions, and on the other hand, the similarity between the amyloid fibrillation process of HEWL and that of A*β* proteins involved in AD, make this protein an ideal option for use in the protein misfolding and aggregation studies. Furthermore, HEWL is a low cost, well-characterized protein whose essential structural information is available in the literature [22,23].

Based on aforementioned considerations, herein we explored the interactions of Ga(DAC)_3_, In(DAC)_3_ and VO(DAC)_2_ with HEWL. Along with the binding process, the effects of these metal complexes on the amyloid fibril formation of HEWL were investigated.

## 2. Experimental

### 2.1. Materials

HEWL (L6876, purity ≥ 90%, activity ≥ 40 000 units/mg), thioflavin T (ThT) (C_17_H_19_ClN_2_S), sodium dihydrogen phosphate (NaH_2_PO_4_), and disodium hydrogen phosphate (Na_2_HPO_4_) were purchased from Sigma-Aldrich and used as received. As reported by Mohammadi *et al*., [19]. Metal diacetylcurcumin complexes used in this work were synthesized. All buffer solutions were prepared with double distilled water.

### 2.2. Preparation of hen egg-white lysozyme stock solution

The preparation of HEWL stock solution was done by dissolving the appropriate amount of the protein in phosphate-buffered saline (PBS) (0.05 M, pH 7.4). The exact concentration of HEWL was determined spectrophotometrically using a Pharmacia Biotech Ultraspec 4000 UV/Vis spectrophotometer at 280 nm (*ε*_280 nm_ = 38 940 M^−1^ cm^−1^) [24].

### 2.3. Preparation of stock solutions of curcumin-based metal complexes

The stock solutions (0.8 mM) of Ga(DAC)_3_, In(DAC)_3_ and VO(DAC)_2_ complexes were prepared by dissolving the appropriate amount of them in dimethylformamide (DMF) solvent. At the end of the dissolution, the colour of the final solutions was transparent yellow. Considering that these metal complexes of diacetylcurcumin are new synthetic compounds whose molar absorption coefficients have not been reported in DMF in the literature, the solutions of them were prepared at first by carefully weighing, and then their molar absorption coefficients were obtained spectrophotometrically using calibration curves. The estimated molar absorption coefficients of Ga(DAC)_3_, In(DAC)_3_ and VO(DAC)_2_ complexes were respectively *ε*_415 nm_ = 11 555 M^−1^ cm^−1^, *ε*_406 nm_ = 109 473 M^−1^ cm^−1^, and *ε*_417 nm_ = 25 368 M^−1^ cm^−1^. After sealing the sample tubes with parafilm and wrapping them with aluminium foil to protect them from evaporation and sunlight, the stock solutions were stored in the refrigerator at 4°C during all experiments.

### 2.4. Preparation of hen egg-white lysozyme amyloid fibrils

To prepare HEWL amyloid fibrils, a solution of the protein (5 ml, 70 µM) was prepared in 100 mM PBS, and the pH adjusted to 2.0 using hydrochloric acid. The solution was then incubated in a shaker incubator bath at 450 rpm and 58°C for 140 min. To investigate the effect of the diacetylcurcumin-based metal complexes on HEWL amyloid fibrillation, three separate samples, each containing HEWL (70 µM) and one of the metal complexes of diacetylcurcumin (10 µM), were prepared and incubated under the above conditions.

### 2.5. Characterization

#### 2.5.1. Intrinsic and synchronous fluorescence spectroscopy

The intrinsic fluorescence spectra of HEWL (3 µM) in the absence and presence of Ga(DAC)_3_, In(DAC)_3_ and VO(DAC)_2_ complexes were collected using a Varian Cary Eclipse fluorescence spectrophotometer equipped with a 10 mm quartz cell by adjusting the excitation wavelength to 295 nm and setting the width of both excitation and emission slits at 5 nm. After adding the mentioned concentrations of the metal complexes of diacetylcurcumin to HEWL solution, the samples were scanned in the wavelength range of 300–500 nm. Corrections were made for the inner filter effect on the fluorescence spectra according to the following equation:

$$F_{\mathrm{cor}}=F_{\mathrm{obs}}\times 10^{(A_{\mathrm{ex}}+A_{\mathrm{em}})/2},$$

where *F*_cor_ is corrected fluorescence intensity, *F*_obs_ is the observed (uncorrected) fluorescence intensity, *A*_ex_ is the absorbance at the fluorescence excitation wavelength and *A*_em_ is the absorbance at the selected fluorescence emission wavelength.

To obtain synchronous fluorescence spectra, simultaneous scanning of excitation and emission monochromators was performed in such a way as to maintain a constant wavelength interval between them. The changes in the molecular microenvironment of tyrosine and tryptophan residues of HEWL were monitored by fixing the wavelength interval (Δ*λ* = *λ*_em_ − *λ*_ex_) individually at 15 and 60 nm, respectively. The concentrations of HEWL and three metal complexes of diacetylcurcumin were the same as those mentioned for the intrinsic fluorescence spectroscopy.

#### 2.5.2. Circular dichroism spectroscopy

The circular dichroism (CD) spectra were recorded on an Aviv spectropolarimeter model 215 (Proterion Corp., USA) equipped with a 10 mm path length cuvette at 25°C. To probe the changes in the tertiary structure of HEWL after interaction with the metal complexes of diacetylcurcumin, the samples were scanned in the near-UV CD (260–340 nm) region. For obtaining the near-UV CD spectra, the buffer contribution from the original protein spectra was subtracted. The visible CD spectra were also obtained by scanning the 350–700 nm region. The induced CD spectra were acquired by subtracting the CD contribution of HEWL from the CD spectrum of the corresponding bioconjugates. All CD spectra were displayed as plots of *θ* (ellipticity) in units of millidegrees (mdeg) versus the scanned wavelength. The concentration of HEWL was 3 µM and two concentrations of the metal complexes of diacetylcurcumin, 1.5 and 3 µM, were used.

#### 2.5.3. Thioflavin T assay

For ThT assay, a 17 µl volume of the incubated HEWL (70 µM) samples was added to 983 µl of ThT solution for the final HEWL and ThT concentrations of 1.2 µM and 20 µM, respectively. ThT spectra containing the incubated HEWL/metal complexes were recorded 5 min after recording the spectrum of ThT solution as control solution. All spectra were recorded using a Varian Cary Eclipse fluorescence spectrophotometer at 25°C. ThT solution was excited with a 440 nm wavelength and emission fluorescence spectra were recorded in the wavelength range of 450–600 nm.

#### 2.5.4. Atomic force microscopy

Atomic force microscopy (AFM) images were obtained using a VEECO-Thermo Microscopes Auto Probe CP Research Atomic Force Microscope in non-contact mode. For AFM analysis, a small volume (approx. 10 µl) of the prepared amyloid samples was dropped on a freshly sliced mica substrate. After 30 min, the mica sheet was washed with 100 µl of deionized water and allowed to dry at room temperature.

## 3. Results and discussion

### 3.1. Interaction of hen egg-white lysozyme with Ga(DAC)_3_, In(DAC)_3_ and VO(DAC)_2_ complexes

#### 3.1.1. Fluorescence quenching study

**Figure 2.**
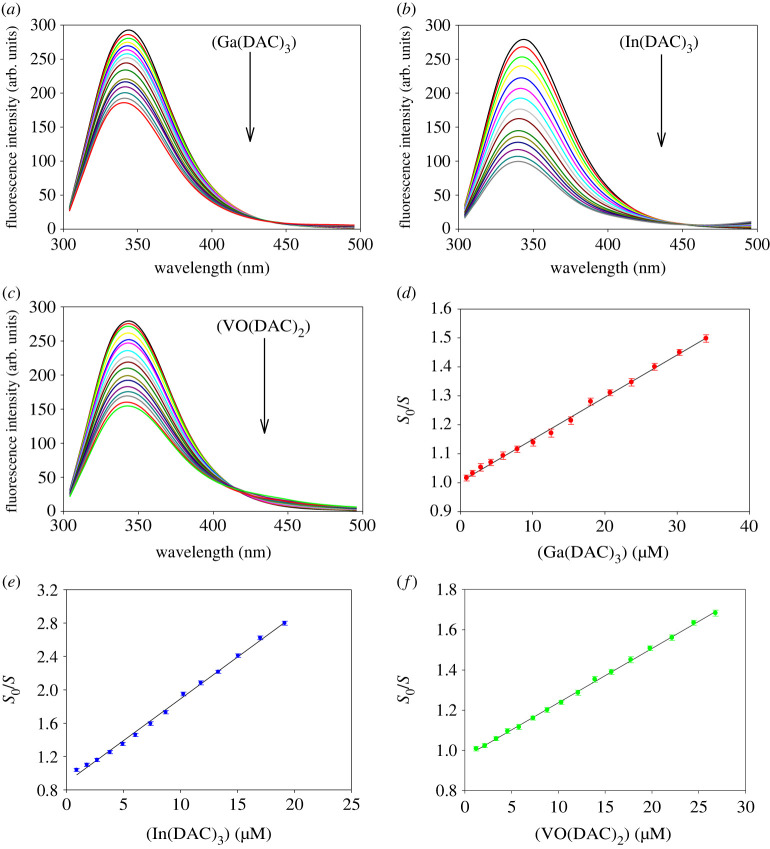
Intrinsic fluorescence spectra of HEWL (3 *μ*M) in the absence and presence of increasing concentrations of (*a*) Ga(DAC)_3_, (*b*) In(DAC)_3_, and (*c*) VO(DAC)_2_ complexes at 293 K. Stern-Volmer plots of (*d*) HEWL/Ga(DAC)_3_, (*e*) HEWL/In(DAC)_3_ and (*f*) HEWL/VO(DAC)_2_ systems at 293 K.

The intrinsic emission fluorescence spectroscopy is a widely used method in studying the fluorescence quenching process of proteins upon interaction with various quenchers, from small molecules to nanoparticles [21,25,26]. Hence, this technique was used to determine the quenching parameters and binding constants of the interaction between HEWL and three metal complexes of diacetylcurcumin, and reveal the thermodynamic nature of the binding processes (figure 2; electronic supplementary material, figures S1, S2, and S3). By excitation at 295 nm, the contribution of tyrosine residues of HEWL in the emission fluorescence is eliminated and the net fluorescence of the protein is emitted only by tryptophan residues of the protein. As evidenced from figures 2*a*–*c*, HEWL has an emission spectrum with a maximum intensity at 345 nm, whose intensity gradually decreases with increasing concentration of the metal complexes of diacetylcurcumin. This observation shows the high quenching ability of these complexes towards tryptophan residues of HEWL. Moreover, the quenching processes are associated with blue shifts of 4, 4 and 2 nm, for Ga(DAC)_3_, In(DAC)_3_ and VO(DAC)_2_ complexes respectively, indicating that the microenvironment of tryptophan residues of HEWL becomes more hydrophobic after interacting with these metal complexes of diacetylcurcumin.

The fluorescence quenching process of HEWL in the presence of three metal complexes of diacetylcurcumin can be quantified according to the well-known Stern-Volmer equation:

3.1 $$\frac{F_{0}}{F}=1+K_{SV}[Q]=1+k_{q}\tau_{0}[Q],$$

where *F*_0_ and *F* represent the intrinsic emission intensities of HEWL fluorophores, respectively, in the absence and presence of the metal complexes of diacetylcurcumin, and [*Q*] is the molar concentration of quencher. *K*_SV_, *k*_q_ and *τ*_0_ denote the Stern-Volmer quenching constant, bimolecular quenching rate constant, and the average lifetime of fluorophore without quencher, respectively. The value of *K*_SV_, as a parameter of the sensitivity of the fluorophore to a quencher, can be estimated from the slope of the linear plot of *F*_0_/*F* as a function of [*Q*] (figure 2*d,e*,*f*; electronic supplementary material, figure S1). To obtain more accurate quantification of fluorescence data, the total area of the emission fluorescence peaks (*S*_0_ and *S*) was applied instead of the emission fluorescence intensities (*F*_0_ and *F*) [21]. The fluorescence quenching phenomenon can be generally divided into two distinct classes: static quenching and dynamic quenching. Owing to the reciprocal and direct correlations between the *K*_SV_ parameter and temperature, respectively, for static and dynamic quenching, the observed trend in the *K*_SV_ value versus temperature can be used to distinguish these mechanisms from each other. In dynamic quenching, an increase in temperature leads to faster diffusion of molecules by which weakly bound nonfluorescent complexes are further dissociated, and the value of *K*_SV_ increases [27]. From the data recorded in table 1, it can be seen that the *K*_SV_ value of HEWL/Ga(DAC)_3_ and HEWL/In(DAC)_3_ systems decreases as temperature increases, while it increases with increasing temperature for the HEWL/VO(DAC)_2_ system. Therefore, it can be concluded that the fluorescence quenching of HEWL in the presence of Ga(DAC)_3_ and In(DAC)_3_ quenchers takes place by means of static mechanism while the fluorescence quenching of the HEWL/VO(DAC)_2_ system is governed by a dynamic mechanism. Considering a lifetime value of 10^−8^ s for the native fluorophores, [27] the equation *k*_q_ = *K*_SV_/*τ*_0_ gives the *k*_q_ values greater than the diffusion-limited quenching value of 2.0 × 10 ^+ 10^ M^−1^ s^−1^ for all systems [28]. Therefore, it can be inferred that the quenching process of all systems occurs in part through a static mechanism. Indeed, the VO(DAC)_2_ complex quenches the emission fluorescence of HEWL by a dual mechanism, including both static and dynamic quenching, while the quenching process of the two other systems proceeds only by a static mechanism.

**Table 1.** Fluorescence quenching parameters of HEWL/Ga(DAC)_3_, HEWL/In(DAC)_3_, and VO(DAC)_2_ bioconjugate systems at different temperatures.

bioconjugate system	*T* (K)	*K*_SV_ (M^−1^)	*k*_q_ (M^−1^ s^−1^)	*n*	*K*_b_ (M^−1^)
HEWL/Ga(DAC)_3_	293	(1.46 ± 0.02) × 10^+4^	(1.46 ± 0.02) × 10^+12^	(0.92 ± 0.02)	(1.11 ± 2.59) × 10^+3^
	303	(1.30 ± 0.01) × 10^+4^	(1.30 ± 0.01) × 10^+12^	(0.80 ± 0.02)	(1.59 ± 0.75) × 10^+3^
	308	(1.25 ± 0.01) × 10^+4^	(1.25 ± 0.01) × 10^+12^	(0.89 ± 0.02)	(3.74 ± 1.69) × 10^+3^
HEWL/In(DAC)_3_	293	(0.10 ± 0.00) × 10^+6^	(0.10 ± 0.00) × 10^+14^	(1.26 ± 0.01)	(1.74 ± 0.54) × 10^+6^
	303	(9.61 ± 0.10) × 10^+5^	(9.61 ± 0.10) × 10^+13^	(1.25 ± 0.04)	(1.79 ± 2.12) × 10^+6^
	308	(1.04 ± 0.02) × 10^+5^	(1.04 ± 0.02) × 10^+13^	(1.25 ± 0.02)	(1.82 ± 1.15) × 10^+6^
HEWL/VO(DAC)_2_	293	(2.70 ± 0.02) × 10^+4^	(2.70 ± 0.02) × 10^+12^	(1.35 ± 0.04)	(1.23 ± 1.48) × 10^+6^
	298	(2.70 ± 0.04) × 10^+4^	(2.70 ± 0.04) × 10^+12^	(1.15 ± 0.03)	(1.40 ± 1.22) × 10^+5^
	303	(2.78 ± 0.03) × 10^+4^	(2.78 ± 0.03) × 10^+12^	(0.93 ± 0.02)	(1.19 ± 0.50) × 10^+4^
	308	(2.80 ± 0.02) × 10^+4^	(2.80 ± 0.02) × 10^+12^	(0.75 ± 0.04)	(1.66 ± 1.78) × 10^+3^

Assuming that all the binding sites of HEWL are identical and independent and an equilibrium interaction is established between HEWL and each of the metal complexes of diacetylcurcumin, the fluorescence quenching process can be further analysed by the following double logarithmic equation:

3.2 $$\log\left(\frac{F_{0}-F}{F}\right)=\log K_{b}+n\log[Q],$$

where *K*_b_ and *n* represent the binding constant and number of binding sites on each HEWL molecule, respectively. Plotting log[(*F*_0_ − *F*)/*F*] versus log[*Q*] produces a straight line whose slope and intercept can be used respectively to estimate the values of *n* and *K*_b_ (electronic supplementary material, figures S2A, S2B, S2C and S3). As recorded in table 1, the values of *n* are approximately close to unity for all systems, indicating that the interaction of HEWL with the metal complexes of diacetylcurcumin is mediated by a single binding site on the protein. In other words, the resulting bioconjugates are the product of the interaction between HEWL and the metal complexes of diacetylcurcumin with a 1 : 1 stoichiometry. On the basis of a great discrepancy between the minimum value of the binding constant (in the order of 10^+3^ M^−1^) for the HEWL/Ga(DAC)_3_ system and its maximum value (in the order of 10^+6^ M^−1^) for the HEWL/In(DAC)_3_ system, it can be inferred that HEWL interacts more strongly with the In(DAC)_3_ complex than the two other metal complexes. By comparison with the reported value of *K*_b_ for interaction of diacetylcurcmin with HEWL (10^+4^ M^−1^) [21], it can be concluded that complexation of three diacetylcurcumin ligands to Ga did not affect the binding affinity towards lysozyme, whereas in the case of In(DAC)_3_ and VO(DAC)_3_ a significant increase in the binding affinity has occurred. So far, the interactions of diacetylcurcumin with different proteins such as lysozyme [21], human and bovine serum albumin [29], bovine β-lactoglobulin [30], ribunclease A [31], β-casein [32], ribunclease A, and bovine α-lactalbumin [33] have been reported, whereas there is no report on the interactions of Ga(DAC)_3_, In(DAC)_3_, and VO(DAC)_2_ with different proteins. The values of binding constants for interactions of diacetylcurcumin with different proteins are listed in the electronic supplementary material, table S1. As shown in this table, the higher binding affinities of diacetylcurcumin were reported towards β-casein and ribonuclease A, while the lowest ones were for human serum albumin and β-lactoglobulin than other proteins.

Moreover, the effect of increasing temperature on the binding affinity of HEWL to the metal complexes of diacetylcurcumin is more pronounced for the HEWL/VO(DAC)_2_ system. As mentioned before, the VO(DAC)_2_ complex reduces the fluorescence intensity of HEWL mainly by dynamic quenching. In this mechanism, the kinetic energy and diffusion rate of molecules increase with increasing temperature, and weakly bound complexes are further dissociated at higher temperatures. Therefore, a three-order decrease in the binding constant value of HEWL to the VO(DAC)_2_ complex (from 10^+6^ to 10^+3^ M^−1^) upon increasing temperature is simply rationalized. By contrast to dynamic quenching, increasing temperature for HEWL/Ga(DAC)_3_ and HEWL/In(DAC)_3_ systems, governed by static quenching, leads to an enhancement in the binding affinity of the protein to the metal complexes by which the resulting nonfluorescent bioconjugates become more stable at higher temperatures (see the *K*_b_ values in table 1).

To disclose the thermodynamic nature of the HEWL/metal complex systems, the van't Hoff equation was used:

3.3 $$\ln K_{b}=-\frac{\Delta H^{0}}{RT}+\frac{\Delta S^{0}}{R},$$

where Δ*H*^0^, Δ*S*^0^, *R* and *T* represent respectively the enthalpy change, the entropy change, the universal gas constant, and absolute temperature, and *K*_b_ denotes the binding constant at the corresponding temperature. A plot of ln*K*_b_ versus 1/T (electronic supplementary material, figures S2D, S2E and S2F) gives a straight line with −Δ*H*^0^/*R* as the slope and Δ*S*^0^/*R* as the intercept. The Gibbs free energy change, Δ*G*^0^, was also estimated using the following equation:

3.4 $$\Delta G^{0}=\Delta H^{0}-T\Delta S^{0}.$$

For HEWL/Ga(DAC)_3_ and HEWL/VO(DAC)_2_ systems, the negative signs of Δ*H*^0^ and Δ*S*^0^ (table 2) indicate that the main forces involved in stabilizing the formed bioconjugates are van der Waals interactions and hydrogen bonds [34]. Also, the negative sign of Δ*G*^0^ combined with the previous two negative signs imply that the interaction between HEWL and two metal complexes of diacetylcurcumin, i.e. Ga(DAC)_3_ and VO(DAC)_2_, occurs by an exothermic, enthalpy-driven and spontaneous process. In the case of the HEWL/In(DAC)_3_ system, the modes of interaction between the protein and In(DAC)_3_ complex are electrostatic interactions as Δ*H*^0^ < 0 and Δ*S*^0^ > 0 (table 2) [34]. Moreover, based on the enthalpy and entropy changes and the negative value of Δ*G*^0^, it can be stated that HEWL binds to the In(DAC)_3_ complex by an exothermic, entropy-driven and spontaneous process. The reported values of thermodynamic functions for interaction of diacetylcurcumin with HEWL revealed that similar to current cases of Ga(DAC)_3_ and VO(DAC)_2_ *ΔH*^0^ and *ΔS*^0^ are negative (−99.04 kJ mol^−1^ and −237.98 J mol^−1^ K^−1^) [21].

**Table 2.** Thermodynamic functions of HEWL/Ga(DAC)_3_, HEWL/In(DAC)_3_, and VO(DAC)_2_ bioconjugate systems at different temperatures.

bioconjugate system	*T* (K)	*ΔH*^0^ (kJ mol^−1^)	*ΔS*^0^ (J mol^−1^ K^−1^)	*ΔG*^0^ (kJ mol^−1^)
HEWL/Ga(DAC)_3_	293	−24.34 ± 0.17	−10.50 ± 0.55	−21.26 ± 0.58
	303	−24.34 ± 0.17	−10.50 ± 0.55	−21.16 ± 0.58
	308	−24.34 ± 0.17	−10.50 ± 0.55	−21.11 ± 0.58
HEWL/In(DAC)_3_	293	−0.62 ± 0.00	117.38 ± 0.01	−35.01 ± 0.01
	303	−0.62 ± 0.00	117.38 ± 0.01	−36.19 ± 0.01
	308	−0.62 ± 0.00	117.38 ± 0.01	−36.77 ± 0.01
HEWL/VO(DAC)_2_	293	−334.67 ± 1.03	−1024.87 ± 3.44	−34.38 ± 3.59
	298	−334.67 ± 1.03	−1024.87 ± 3.44	−29.26 ± 3.59
	303	−334.67 ± 1.03	−1024.87 ± 3.44	−24.13 ± 3.59
	308	−334.67 ± 1.03	−1024.87 ± 3.44	−19.01 ± 3.59

#### 3.1.2. Determination of binding distances

Förster resonance energy transfer (FRET) is a sensitive fluorescence-based approach that acts as a spectroscopic ruler to determine the binding distances within 1–10 nm between interacting molecules [27,35,36]. Owing to the good overlap between the emission fluorescence spectrum of HEWL, as donor molecule, and the absorption spectra of three metal complexes of diacetylcurcumin, as acceptor molecules (electronic supplementary material, figure S4), the FRET method was used to obtain distance-dependent information of the donor-acceptor pairs. According to Förster's non-radiative energy transfer theory [27], the value of energy transfer efficiency, E, can be measured using the following equation:

3.5 $$E=1-\frac{F}{F_{0}}=\frac{R_{0}^{6}}{R_{0}^{6}+r^{6}},$$

where *R*_0_ and *r* represent the critical energy transfer distance and distance between donor and acceptor, respectively. The *R*_0_ parameter, the distance for 50% energy transfer, can be also calculated by the following equation:

3.6 $$R_{0}^{6}=8.79\;\times \;10^{-25}[K^{2}n^{-4}\phi J(\lambda )],$$

where *K*^2^ is the relative orientation of the donor and acceptor (equal to 2/3 for a random orientation), *n* denotes the average refractive index of the medium (equal to 1.33), and *φ* represents the fluorescence quantum yield of the donor (equal to 0.15). *J*(λ), a parameter describing the donor–acceptor spectral overlap, can be estimated according to the following equation:

3.7 $$J(\lambda )=\frac{\int_{0}^{\infty }F_{D}(\lambda )\varepsilon_{A}(\lambda )\lambda^{4}d\lambda }{\int_{0}^{\infty }F_{D}(\lambda )d\lambda },$$

where *F*_D_(λ) and *ε*_A_ are the emission fluorescence intensity of the donor and the molar absorption coefficient of the acceptor at respective wavelength *λ*, respectively. The electronic supplementary material, table S2 lists the values obtained of the binding-related parameters, namely, *J*(λ), *R*_0_, *E* and *r* for the systems under study. According to the data recorded in the electronic supplementary material, table S2, the *r* values for all systems are considerably smaller than 8 nm, so that two conditions of a high-efficiency FRET process between a donor-acceptor pair, *r* < 8 nm and 0.5*R*_0_ < *r* < 1.5*R*_0_ [37], are well satisfied. Therefore, it can be concluded that a non-radiative energy transfer process contributes to the fluorescence quenching of HEWL by the metal complexes of diacetylcurcumin. Moreover, the least value of the binding distance is observed for the HEWL/In(DAC)_3_ bioconjugate, indicating that HEWL interacts more strongly with the In(DAC)_3_ complex than the two other complexes. This observation matches well with the fluorescence quenching results (see the *K*_b_ values in table 1). In the case of the HEWL/In(DAC)_3_ bioconjugate, a relatively high energy transfer value of 18% indicates that a non-radiative energy transfer is substantially involved in the fluorescence quenching of HEWL by the In(DAC)_3_ complex. On the other hand, the contribution of this process to the fluorescence quenching of two other bioconjugates is very low, reaching about 5%. The reported FRET calculation of HEWL/DAC showed binding distance of less than 2 nm (1.81 nm) [21] indicating that more expanded spatial structure of diacetylcurcumin complexes in comparison to one diacetylcurcumin molecule may impede closer binding to the appropriate site of the protein.

#### 3.1.3. Conformational analysis of hen egg-white lysozyme

##### 3.1.3.1. Synchronous fluorescence spectroscopy

To investigate the effect of the metal complexes of diacetylcurcumin on the conformation of tyrosine and tryptophan residues of HEWL, synchronous fluorescence spectra of HEWL were recorded alone and in the presence of the metal complexes (electronic supplementary material, figure S5). By fixing the Δ*λ* value, i.e. the difference between excitation and emission wavelengths, at 15 and 60 nm, synchronous fluorescence spectroscopy provides useful information on the microenvironmental changes of tyrosine and tryptophan residues of proteins, respectively [38]. As shown in the electronic supplementary material, figure S5, all three metal complexes of diacetylcurcumin strongly quench the emission fluorescence of HEWL at both 15 and 60 nm. Moreover, the observation of a greater quenching in the emission fluorescence of tryptophan residues (Δ*λ* = 60 nm) compared to tyrosine residues (Δ*λ* = 15 nm) indicates that the metal complexes of diacetylcurcumin are probably closer to tryptophan residues [39]. At Δ*λ* = 15 nm, no shift in the emission maximum of HEWL is observed, whereas at Δ*λ* = 60 nm, a 2 nm blue shift is observed in the emission spectrum of tryptophan residues of the protein after the interaction with the metal complexes of diacetylcurcumin except for the VO(DAC)_2_ complex. Thus, it can be inferred that the conformation of tyrosine residues of HEWL remains intact by interaction with three quenchers, while Ga(DAC)_3_ and In(DAC)_3_ complexes make the microenvironment of tryptophan residues of HEWL more hydrophobic. In the case of VO(DAC)_2_, the polarity of the microenvironment surrounding tryptophan residues of HEWL remains unaffected as the quencher approaches them. Overall, it can be concluded that the formation of HEWL/metal complex bioconjugates is associated with the binding of three metal complexes of diacetylcurcumin to both tyrosine and tryptophan residues in which the conformational changes can occur only around the tryptophan residues of the protein. It should be noted that the reduction in the polarity of the microregions surrounding tryptophan residues of the protein has already been observed by the intrinsic emission fluorescence titrations (see §3.1.1.).

##### 3.1.3.2. Circular dichroism spectroscopy

**Figure 3.**
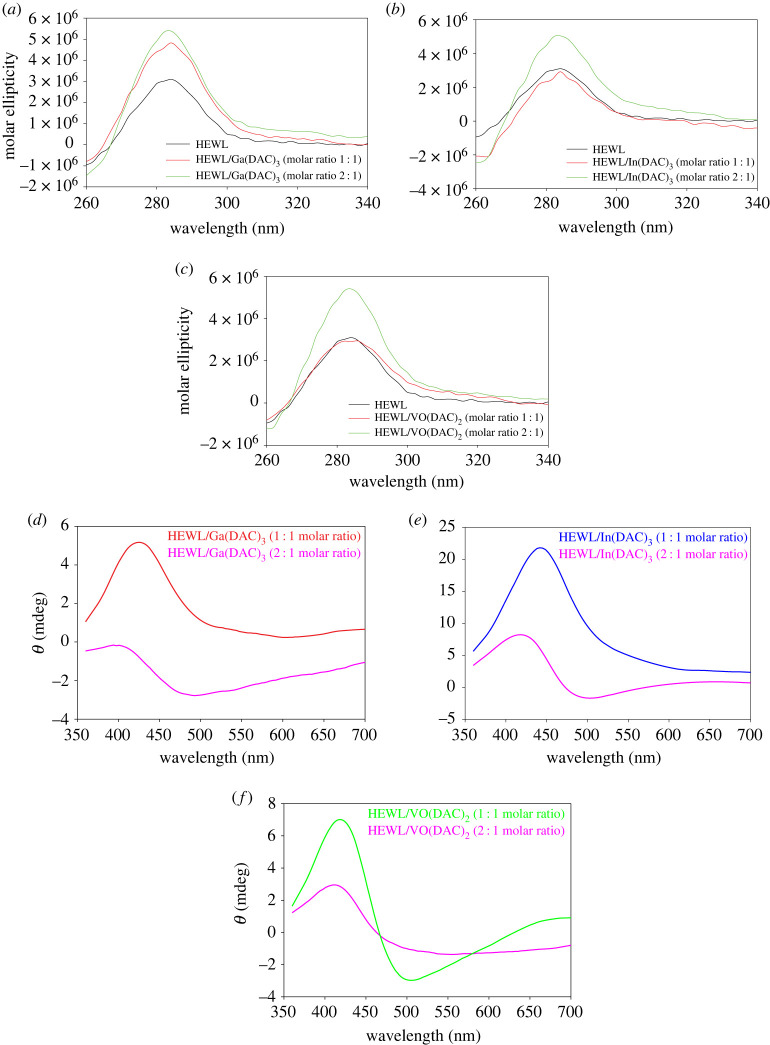
Near-UV CD spectra of HEWL (40 µM) at 2 : 1 and 1 : 1 molar ratios of the protein to (*a*) Ga(DAC)_3_, (*b*) In(DAC)_3_ and (*c*) VO(DAC)_2_ complexes. Visible CD spectra of (*d*) Ga(DAC)_3_, (*e*) In(DAC)_3_ and (*f*) VO(DAC)_2_ complexes at molar ratios of 1 : 2 and 1 : 1 to HEWL.

To detect the alterations in the tertiary structure of HEWL upon interacting with the metal complexes of diacetylcurcumin, the near-UV CD (260–340 nm) spectra of HEWL alone and the formed bioconjugates were recorded (figure 3*a*–*c*). According to figure 3, the near-UV CD spectrum of HEWL consists of a relatively broad positive peak centred at 283 nm attributed to the aromatic amino acid residues of the protein [40], whose intensity shifts towards more positive values with increasing concentration of the metal complexes of diacetylcurcumin. The less intense negative signal in the range of 260–270 nm as well as a broad positive peak centred at 283 nm are in accordance with reported near-UV CD spectra of native lysozyme [41,42]. It can be inferred that after interacting with the metal complexes of diacetylcurcumin, HEWL acquires a more compact folded tertiary structure with higher rigidity in which aromatic groups have more contact either with one another or with the other groups of the protein molecule [43]. Moreover, since the overall shape of the CD spectrum of free HEWL is the same as that of the formed bioconjugates, it can be concluded that the metal complexes of diacetylcurcumin are not able to cause major conformational changes in the tertiary structure of the protein.

In the visible CD (350–700 nm) region, asymmetric proteins like HEWL by making chiral changes in the structure of achiral ligands like our metal complexes of diacetylcurcumin create their induced CD spectra. The induced CD bands originated from conformational adaptation of ligands relative to asymmetric protein binding sites are called Cotton effects (CEs). The visible CD spectra of the metal complexes of diacetylcurcumin at 1 : 2 and 1 : 1 molar ratios of metal complex : HEWL are shown in figure 3*d*–*f*. At a 1 : 2 molar ratio of Ga(DAC)_3_ to HEWL, the visible CD spectrum of the metal complex consists of a positive CE and a negative CE, which are centred at 400 and 490 nm, respectively. A similar behaviour is observed for the In(DAC)_3_ complex but with the difference that its positive CE is located at about 420 nm. A positive CE at shorter wavelengths followed by a negative CE at longer wavelengths indicates that the conformer adopts an anticlockwise chirality or M-helicity configuration [44]. When the concentration of Ga(DAC)_3_ and In(DAC)_3_ becomes equal to that of HEWL, the negative Cotton bands disappear and a positive Cotton peak with higher intensity at around 425 nm for Ga(DAC)_3_ and 440 nm for In(DAC)_3_ emerges. Therefore, it can be concluded that the chiral configuration of Ga(DAC)_3_ and In(DAC)_3_ complexes in the binding site of the protein is the same. In the case of the VO(DAC)_2_ complex, there is only one single positive Cotton band centred at 410 nm when the molar ratio of the complex to HEWL is 1 : 2. At a 1 : 1 molar ratio of VO(DAC)_2_ to HEWL, in addition to an intense positive Cotton band at around 418 nm, a new intense negative Cotton band at around 503 nm appears, indicating that HEWL induces an anticlockwise chirality in the structure of the VO(DAC)_2_ complex. Our previous report on the interaction of diacetylcurcumin with HEWL showed different induced CD spectra [21] indicating that chairality induced by the protein binding site is different for diacetylcurcumin and its metal complexes.

Overall, synchronous fluorescence and CD experiments demonstrate that the formation of the bioconjugates is associated with a minor conformational change in the microenvironment of tryptophan residues, not in the whole structure of the protein, and induction an anticlockwise chirality in the structure of the metal complexes of diacetylcurcumin.

### 3.2. Antiamyloidogenic activity of Ga(DAC)_3_, In(DAC)_3_ and VO(DAC)_2_ complexes towards hen egg-white lysozyme fibril formation

#### 3.2.1. Thioflavin T fluorescence assay

**Figure 4.**
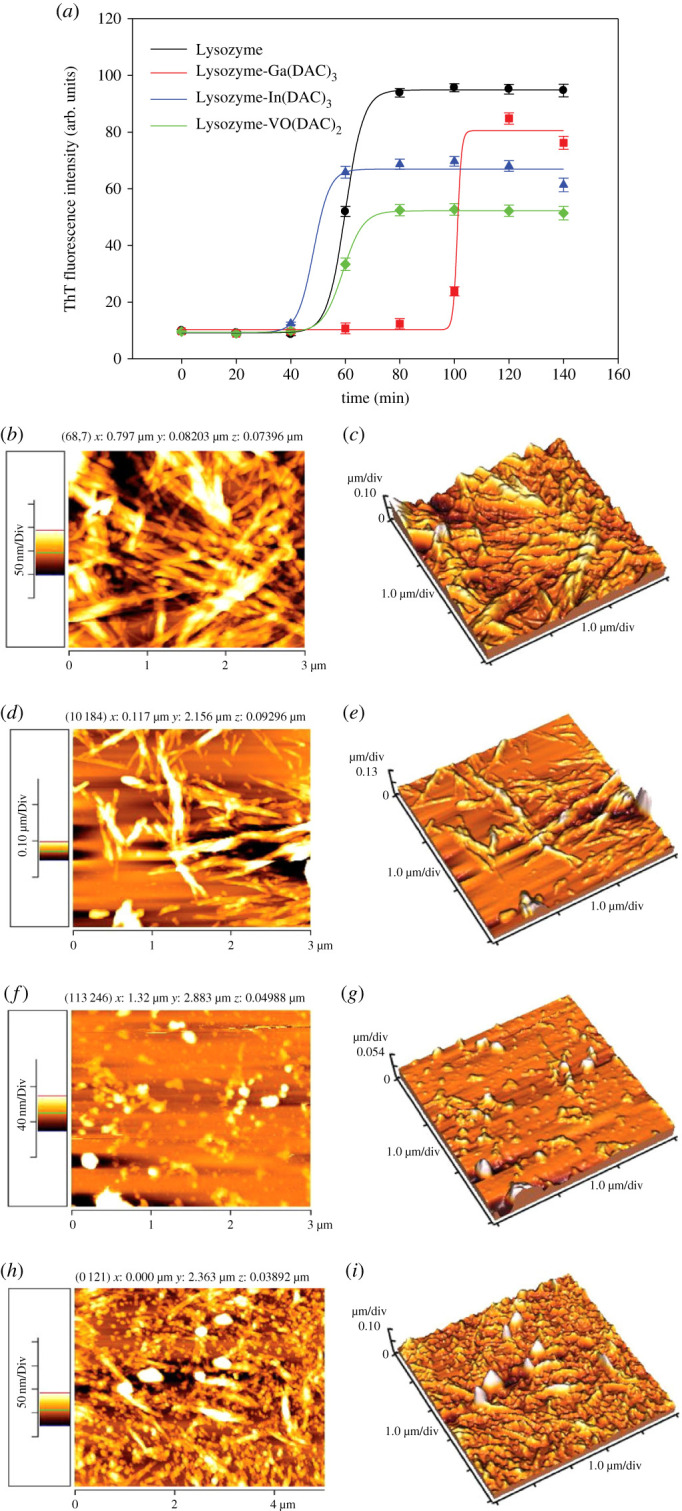
(*a*) Kinetics of fibrillation of HEWL (70 µM) at pH 2.0, in the absence and presence of Ga(DAC)_3_, In(DAC)_3_, and VO(DAC)_2_ complexes. AFM images (left column) along with their corresponding topographic maps (right column) of HEWL (70 µM) alone (*b* and *c*), and in the presence of Ga(DAC)_3_ (*d* and *e*), In(DAC)_3_ (*f* and *g*), and VO(DAC)_2_ (*h* and *i*) incubated under amyloid-forming conditions (pH 2, 54°C, 150 rpm). The concentarion of metal complexes is 10 µM.

Enhancement of ThT fluorescence intensity upon its rapid and strong binding to the cross β-sheet structure of amyloid fibrils is important evidence to confirm the amyloid fibril formation of proteins. The inhibitory activities of Ga(DAC)_3_, In(DAC)_3_ and VO(DAC)_2_ complexes on the amyloid fibrillation process of HEWL were explored by ThT fluorescence assay over 140 min period of incubation at pH 2.0. The kinetics of the fibrillation process in the absence and presence of the considered complexes is depicted in figure 4*a.* As can be seen in this figure, all three complexes have decreased the maximum emission of ThT indicating their antiamyloidogenic activities. The VO(DAC)_2_ showed the highest inhibitory effect, while In(DAC)_3_ exhibited a considerable delaying effect on the fibrillation process.

To estimate the lag time and apparent rate constant for growth of fibrils, the following equation was employed on the ThT fluorescence data [45]:

$$F=F_{\min}+\frac{F_{\max}}{1+e^{-(t-t_{0}/\tau )}},$$

where *F* is the fluorescence intensity in *t*, *F*_min_ and *F*_max_ are fluorescence intensities in initial time and saturation phase, respectively, *t* is incubation time and *t*_0_ is required time to get 50% of maximal fluorescence intensity. The apparent rate constant, *k*_app_, is given by 1/*τ* and the lag time is approximated by *t*_0_ − 2*τ*. The kinetic parameters extracted from the curve fitting to sigmoidal equation are presented in the electronic supplementary material, table S3. As can be seen in this table, amyloid fibrillation process of HEWL in the presence of In(DAC)_3_ was accompanied by a significant increase in the lag time and *t*_0_. The higher delaying effect of In(DAC)_3_ and inhibitory effect of VO(DAC)_2_ on the fibril formation of HEWL can be attributed to the higher binding affinities of In(DAC)_3_ and VO(DAC)_2_ towards HEWL compared to Ga(DAC)_3_ (see the *K*_b_ values in table 1). Our previous report about the reported delaying and inhibitory effect of the diacetylcurcumin molecule on the amyloid fibrillation of HEWL [21] along with the current results indicate that the diacetylcurcumin as ligand in the chemical structure of the considered metal complexes contributes to the observed inhibiting activities against amyloid fibril formation of HEWL.

#### 3.2.2. Morphology analysis by atomic force microscopy

The AFM imaging was used to explore the morphology of fibrillar structures of HEWL in the absence and presence of Ga(DAC)_3_, In(DAC)_3_ and VO(DAC)_2_ complexes (figure 4*b–i*). As can be seen in figure 4, the mature fibrils of HEWL in the absence of the complexes have been formed under the amyloidogenic conditions. Incubation of HEWL with each of the complexes resulted in decreasing the number and length of the amyloid fibrils confirming the results of ThT fluorescence assay.

## 4. Conclusion

Although there are diverse published reports on the biological activities of curcumin, its limited stability and rapid metabolism in the gastrointestinal tract and limited usage in clinical applications encouraged researchers to develop different metal complexes of curcumin and its analogues. Based on the reported antiamyloidogenic activities of curcumin and diacetylcurcumin, the current work aimed, for the first time to our knowledge, to investigate the inhibitory effects of Ga(DAC)_3_, In(DAC)_3_ and VO(DAC)_2_ complexes against amyloid fibril formation of HEWL as a model protein. Furthermore the details of binding interactions of Ga(DAC)_3_, In(DAC)_3_ and VO(DAC)_2_ complexes with HEWL have been demonstrated. The results of fluorescence quenching at different temperatures revealed the stronger binding affinities of In(DAC)_3_ and VO(DAC)_2_ than Ga(DAC)_3_ towards HEWL. By comparison of the current results with those of the HEWL/DAC bioconjugate, it can suggested that complexation of diacetylcurcumin with indium and oxovanadium resulted in stronger binding interaction with HEWL. The conformational investigations by synchronous fluorescence and near-UV CD spectroscopies showed that formation of HEWL/complex bioconjugates were not accompanied by considerable alteration in the tertiary structure of the protein. The results of the ThT fluorescence experiments indicated the antiamyloidogenic activities of these diacetylcurcumin metal complexes. The higher delaying effect of In(DAC)_3_ and inhibitory effect of VO(DAC)_2_ on the fibril formation of HEWL can be attributed to the higher binding affinities of In(DAC)_3_ and VO(DAC)_2_ towards HEWL compared to Ga(DAC)_3_. For future works, complementary insights into structural conversions involved in early stage oligomers, pre-fibril species and intermediates during amyloid fibrillation of HEWL in the presence of these metal complexes of diacetylcurcumin can be explored by the efficient and reproducible screening techniques such as electrospray ionization mass spectrometry and X-ray crystallography. The reported anti-cancer and anti-oxidant properties of Ga(DAC)_3_, In(DAC)_3_, and VO(DAC)_2_ complexes along with their inhibitory effects on the amyloid fibrillation observed in the current work, can provide motivation to study more aspects and relevant issues such as stability in serum, mechanism of action and potential targets of these metal complexes as future candidates in drug formulation against the diseases originated from the toxic amyloid fibrils in different tissues and organs of body.

## Data Availability

The datasets supporting this article have been uploaded as part of the electronic supplementary material [46].
